# Influence of Cu Content on Precipitation Behavior and Mechanical Properties Under Aging Treatment of Al-Cu-Li Alloys

**DOI:** 10.3390/ma18102172

**Published:** 2025-05-08

**Authors:** Pengcheng Chen, Xiwu Li, Haitao Lin, Kai Wen, Ying Li, Shuyan Wang, Chenyang Xun, Changlin Li, Lizhen Yan, Yongan Zhang, Baiqing Xiong

**Affiliations:** 1 State Key Laboratory of Nonferrous Structural Materials, China GRINM Group Co., Ltd., Beijing 100088, China; chenpc96@163.com (P.C.); liying@grinm.com (Y.L.); m15671688995@163.com (C.X.); lichanglin97@163.com (C.L.); yanlizhen@grinm.com (L.Y.); zhangyongan@grinm.com (Y.Z.); xiongbq@grinm.com (B.X.); 2 GRIMAT Engineering Institute Co., Ltd., Beijing 101407, China; 3 General Research Institute for Nonferrous Metals, Beijing 100088, China; 4 Southwest Aluminium (GROUP) Co., Ltd., Chongqing 401326, China; grinmskl_al@163.com (H.L.); swaalli@126.com (S.W.)

**Keywords:** Al-Cu-Li alloys, Cu content, precipitation behavior, aging treatment

## Abstract

The influence of Cu content (3.10, 3.50, and 3.80 wt.%) on the precipitation behavior and mechanical properties of Al-Cu-Li alloys under two aging conditions (direct aging at 175 °C vs. 3.5% pre-stretching followed by aging at 155 °C) was systematically investigated. The alloys were characterized using hardness testing, tensile property evaluation, and transmission electron microscopy (TEM) to correlate microstructural evolution with performance. The results revealed that increased Cu content accelerated early-stage hardening kinetics and elevated peak hardness and strength. Aging at 175 °C/36 h produced T_1_ phase-dominated microstructures with θ′ phases. With the increase of Cu content, the enhancement effect on the precipitation of T_1_ and θ′ phases becomes more pronounced, gradually overshadowing the initial promotion effect on precipitate growth. Pre-deformation prior to 155 °C/36 h aging induced significant T_1_ phase refinement and proliferation, with increasing Cu content continuously reducing T_1_ phase sizes while moderately enlarging θ′ precipitates. Precipitation-strengthening analysis revealed a transition in T_1_ strengthening from bypass to shearing dominance under 155 °C/36 h aging after pre-deformation, enhanced by Cu-promoted T_1_ refinement, which collectively drove superior strength in high-Cu alloys. These findings provide valuable insights for the composition design and mechanical property optimization of Al-Cu-Li alloys.

## 1. Introduction

Al-Li alloys have been widely utilized in aerospace applications due to their low density and exceptional mechanical properties that rival conventional Al alloys [1,2]. Al-Cu-Li system alloys, enhanced by microalloying elements such as Mg and Ag, currently represent the most extensively applied and high-performance variants [3,4,5]. Their strengthening phases primarily include the T_1_ phase (Al_2_CuLi), θ′ phase (Al_2_Cu), S′ phase (Al_2_CuMg), δ′ phase (Al_3_Li), and β′ phase (Al_3_Zr) [3,4,5,6]. The T_1_ phase exhibits the most pronounced strengthening effect, characterized by its plate-shaped morphology precipitated on {111}_Al_ planes with a semi-coherent interface to the matrix [7,8]. However, the coexistence of multiple phases complicates the precipitation behavior, particularly due to competitive precipitation interactions between some of them [3,9]. Consequently, controlling aging precipitation processes to optimize mechanical performance remains a critical research focus in the development of Al-Cu-Li alloys.

As a primary alloying element, Cu directly participates in the formation of multiple phases, making its content a critical determinant of the final mechanical properties. Sun et al. [10] investigated Al-(2.0–6.5)Cu-1Li-0.4Mg-1Zn-0.1Zr (wt.%, hereafter) alloys and found that at lower Cu content (2.0 wt.%), only T_1_ phases were observed. Increasing Cu content to 4.5 wt.% and 6.5 wt.% resulted in the coexistence of T_1_ and θ′ phases. Below 4.5 wt.%, the yield strength increased with Cu content, but further increases led to a strength reduction in the alloys. Ning et al. [11] investigated the mechanical properties of Al-Cu-1.1Li alloys with varying Cu contents subjected to 5% pre-deformation, followed by 150 °C/38 h aging treatment. The results indicated yield strengths of 528 MPa, 562 Mpa, and 585 MPa for the 3.70, 4.10, and 4.30 wt.% Cu alloys, respectively. Li et al. [12] examined Al-(3.2–3.8)Cu-1.0Li alloys under 6% pre-deformation and 160 °C aging, identifying T_1_ and θ′ phases as the dominant precipitates. While Cu elevation marginally improved strength (550–578 MPa), it significantly reduced elongation. These findings collectively demonstrate that increasing Cu content below a critical threshold enhances strength, though the magnitude of improvement depends on specific alloy compositions and processing conditions. This highlights the necessity for tailored optimization of Cu content to balance strength and ductility in Al-Cu-Li alloys.

Beyond compositional factors, the effects of processing conditions on the microstructure and properties of alloys are equally critical. The selection of aging treatment parameters plays a decisive role in determining precipitation behavior. Two primary aging approaches are commonly employed for Al-Cu-Li alloys: direct artificial aging and pre-deformation followed by aging treatment. Direct aging treatment typically requires elevated temperatures to ensure sufficient thermal activation for precipitate nucleation and growth, while pre-deformation introduces abundant nucleation sites that significantly promote the precipitation of the T_1_ phase [13,14]. Cui et al. [15] investigated microstructure–property evolution during direct aging (155–185 °C) of Al-4.0Cu-0.9Li-0.5Mg-0.4Ag alloy, revealing peak hardness at 72 h for 155 °C aging, which was substantially slower than the 20 h requirement at 185 °C. Xu et al. [16] demonstrated that Al-3.5Cu-1.0Li-0.3Mg-0.4Ag-0.3Mn alloy required 128 h at 150 °C to reach peak strength, while equivalent yield strength was achieved through 4% pre-deformation followed by 150 °C/12 h aging. Research [17] demonstrated that applying 3% pre-deformation prior to 155 °C/24 h aging in an Al-3.5Cu-0.9Li alloy reduced the average T_1_ phase diameter by ~30 nm compared to direct aging, resulting in a 60 MPa yield strength increase and 2% ductility improvement. Similarly, Xie et al. [18] reported that 7% pre-deformation combined with 160 °C/24 h aging generated finer T_1_ precipitates, achieving a 200 MPa higher yield strength than direct aging under equivalent conditions, though with significant ductility loss. Most studies [17,19,20] indicate that pre-deformation followed by aging achieves a better balance between strength and ductility. However, direct aging remains advantageous for applications with lower performance requirements or complex geometries. In summary, relying on a single aging method is insufficient to comprehensively understand the correlation between microstructure and properties in Al-Cu-Li alloys.

In the aforementioned studies, while numerous investigations have simultaneously addressed composition and processing parameters, the research focus on precipitation behavior has predominantly centered on the T_1_ phase. The presence and influence of other phases have often been overlooked, and systematic studies integrating comprehensive strengthening analyses remain relatively scarce. Accordingly, the present study investigated the precipitation behavior and mechanical properties of alloys with different Cu contents (3.10, 3.50, and 3.80 wt.%) under various aging conditions. The research methodology included hardness testing, tensile property evaluation, and TEM microstructural analysis. Furthermore, through quantitative statistical analysis and strengthening calculations, the correlation between precipitates and strength was established, and the strengthening mechanisms under different conditions were analyzed. The findings provide valuable insights for optimizing the composition, microstructure, and properties of high-performance Al-Cu-Li alloys.

## 2. Materials and Methods

The chemical compositions of the experimental alloys are presented in Table 1 and were determined using inductively coupled plasma atomic emission spectroscopy (ICP-AES, Waltham, MA, USA), with triplicate measurements conducted. Average values were subsequently calculated under the condition of maintaining measurement deviations within 5%. The alloys were prepared from pure Al, Cu, Li, Mg, Ag, and Zn ingots and Mg-30Zr and Al-10Mn master alloys. The raw materials were melted in a resistance furnace (SYTYDL, Shenyang, China) at 730 °C and poured into a copper mold under the Ar atmosphere. Subsequently, the as-cast ingot was homogenized for 495 °C/24 h + 515 °C/24 h and cooled to room temperature. Hot extrusion after homogenization was conducted at 460 °C to produce the 16 mm thick plates. Following solution treatment at 520 °C for 2 h, the plates were divided into two groups. One group was directly subjected to aging treatment at 175 °C for 0–216 h. The remaining group underwent 3.5% pre-stretching followed by aging at 155 °C for 0–216 h. For direct aging at 175 °C, the 16 mm thick plates were pre-sectioned along the extrusion direction before solution treatment to facilitate multi-duration aging studies. For the 155 °C aging protocol, 500 mm long plates underwent solution treatment and pre-stretching prior to sectioning, ensuring deformation uniformity. After artificial aging, hardness and tensile specimen machining was conducted. Notably, as-quenched (175 °C/0 h) specimens were fully machined before solution treatment to enable immediate testing after quenching. The selection of aging temperatures and pre-deformation levels was based on the comprehensive analysis of precipitation behavior studies in Al-Cu-Li alloys [10,11,12,13,14,15,16,17,18,19,20], aiming to achieve optimal phase formation and superior mechanical properties. In this study, the alloys were designated according to their Cu content, specifically the 3.10 Cu, 3.50 Cu, and 3.80 Cu alloy.

Samples for mechanical properties testing and microstructure observation were taken from the center-thickness region of the plates along the extrusion direction. Vickers hardness was determined using a Wilson VH1150 tester (Buehler, Lake Bluff, IL, USA) with a load of 5 kg and a dwell time of 15 s, with final values calculated as the average of seven discrete measurements. The pre-stretching deformation was conducted using a WAW-1000 tester (Hualong, Shanghai, China) at a constant displacement rate of 1 mm/min. Tensile properties were evaluated by a CMT4303 machine (MTS, Eden Prairie, MN, USA) equipped with an Epsilon 3542 extensometer with a gauge length of 25 mm. The tensile testing rate was set at 2 mm/min, and the gauge length and diameter of the cylindrical tensile specimen were 25 mm and 5 mm, respectively. Tensile data were derived from averaged results of two specimen sets. X-ray diffraction (XRD, Bruker, Karlsruhe, Germany) was conducted using a Bruker D8 Advance X-ray diffractometer with Cu-Kα radiation for the calculation of dislocation density, with scanning parameters of 2θ spacing of 10–90° and an angular velocity of 3°/min.

The observation of microstructures was performed using a Talos F200X G2 transmission electron microscope (TEM, Thermo Fisher Scientific, Waltham, MA, USA) operated at 200 KV. The precipitation behavior was described using bright-field images, selected-area electron diffraction (SAED) patterns, and high-resolution TEM (HRTEM) images. The samples were mechanically ground to 50 μm and then twin-jet electropolished in a solution of 30% nitric acid and 70% methanol at 16 V and −30 °C. The diameter and thickness of the precipitates were measured from at least three TEM images through the Image-Pro Plus software (6.0, Media Cybernetics, Rockville, MD, USA).

## 3. Results

### 3.1. Artificial Age-Hardening Behavior

**Figure 1:**
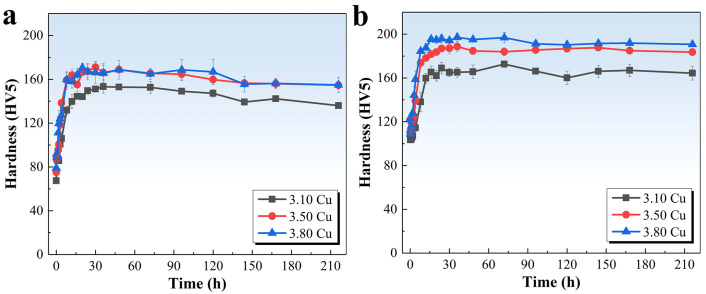
Hardness curves of the alloys with different Cu contents during various conditions: (**a**) 175 °C aging; (**b**) 3.5% pre-deformation followed by aging at 155 °C.

Figure 1 presents the hardness evolution curves of the three alloys under different aging conditions, where Figure 1a corresponds to direct aging at 175 °C, and Figure 1b shows aging at 155 °C after 3.5% pre-deformation. As observed in Figure 1a, the alloys exhibited their minimum hardness values in the as-quenched state, measuring 67 HV5, 75 HV5, and 79 HV5 for the 3.10 Cu, 3.50 Cu, and 3.80 Cu alloys, respectively. A rapid hardness increase occurred within the initial 8 h, with the 3.10 Cu alloy demonstrating a significantly lower hardening rate than the other two. Beyond 8 h, the hardening rate progressively decreased, with peak hardness values of 153 HV5, 171 HV5, and 171 HV5 achieved at 36 h, 30 h, and 20 h for the three alloys, respectively. Subsequent aging led to gradual hardness decline. These results demonstrate that increasing Cu content accelerates the aging response kinetics during 175 °C aging treatment. The hardness increase was most pronounced when raising Cu content from 3.10 wt.% to 3.50 wt.%, while the difference in peak hardness between 3.50 Cu and 3.80 Cu alloys showed negligible improvement.

As shown in Figure 1b, the hardness during initial aging demonstrated an initial decline followed by an ascending trend, with comparable hardness values observed at 0 h and 2 h. Rapid hardening occurred between 2–8 h, followed by a decelerated growth rate. Peak hardness values of 173 HV5, 189 HV5, and 197 HV5 were attained at 72 h, 36 h, and 36 h, respectively, with subsequent minor softening observed. Elevated Cu content correlated with accelerated hardening kinetics during early aging stages and significantly enhanced peak hardness levels. Compared to 175 °C aging treatments, all three alloys exhibited enhanced hardness at equivalent aging durations, extended time-to-peak, and moderated post-peak hardness degradation. Notably, the hardness differential among the three alloys became magnified.

### 3.2. Mechanical Properties

**Figure 2:**
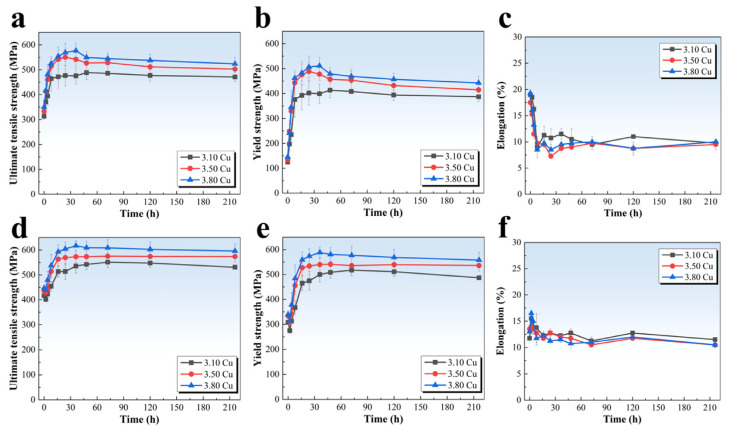
Tensile properties of the alloys during 175 °C aging (**a**–**c**) and 3.5% pre-deformation followed by aging at 155 °C (**d**–**f**): (**a**,**d**) ultimate tensile strength; (**b**,**e**) yield strength; (**c**,**f**) elongation.

Figure 2a–c present the tensile property curves of the alloys during aging at 175 °C. It can be found that the variation trends of ultimate tensile strength (UTS) and yield strength (YS) are approximate to the hardness curves. The initial strength of the alloys was relatively low, with UTS ranging from 314 MPa to 351 MPa and YS between 125 MPa and 146 MPa. With prolonged aging time, all three alloys reached peak strength within 24–48 h. Notably, the 3.10 Cu alloy exhibited significantly lower peak strength (UTS = 489 MPa, YS = 414 MPa) compared to the 3.80 Cu alloy (UTS = 577 MPa, YS = 511 MPa). These findings confirm that increasing Cu content substantially enhances the peak strength of the alloys. In contrast to the strength characteristics, the elongation exhibited an initial decrease followed by a slight increase. After a comprehensive evaluation, the peak aging condition of 175 °C/36 h was selected as the optimal regime for subsequent precipitate characterization and comparison.

Figure 2d–f show the tensile property curves of alloys subjected to 3.5% pre-deformation followed by aging at 155 °C. A distinct hardness and strength reduction was observed during the initial aging stage (within 2 h), followed by subsequent elevation and stabilization after reaching the peak point. The minimum strength values (at 2 h) remained comparable across alloys, with UTS ranging from 402 MPa to 438 MPa and YS between 275 MPa and 314 MPa. Furthermore, peak strength demonstrated significant enhancement with increasing Cu content, as evidenced by the 3.10 Cu alloy (UTS = 551 MPa, YS = 518 MPa) exhibiting over 60 MPa lower peak strength compared to the 3.80 Cu alloy (UTS = 617 MPa, YS = 589 MPa). Notably, the strength differential between peak and minimum values narrowed compared to the 175 °C aging treatment. Contrasting with the strength behavior, elevated Cu content induced a reduction in elongation, though the differences remained statistically insignificant.

In contrast to the 175 °C aging treatment, a time interval exists between the pre-deformation and aging at 155 °C, during which the alloys undergo natural aging. Combined with the dislocation strengthening effect induced by pre-deformation, this results in substantially enhanced hardness and strength at the initial stage compared to the as-quenched condition. Previous studies [21,22] suggest that such strengthening effects gradually diminish during early artificial aging, accounting for the observed decline in hardness and strength. Additionally, the time to reach peak strength was similar for all three alloys, leading to the selection of 155 °C/36 h as the aging regime for subsequent precipitation investigation.

### 3.3. Precipitation Characterization

**Figure 3:**
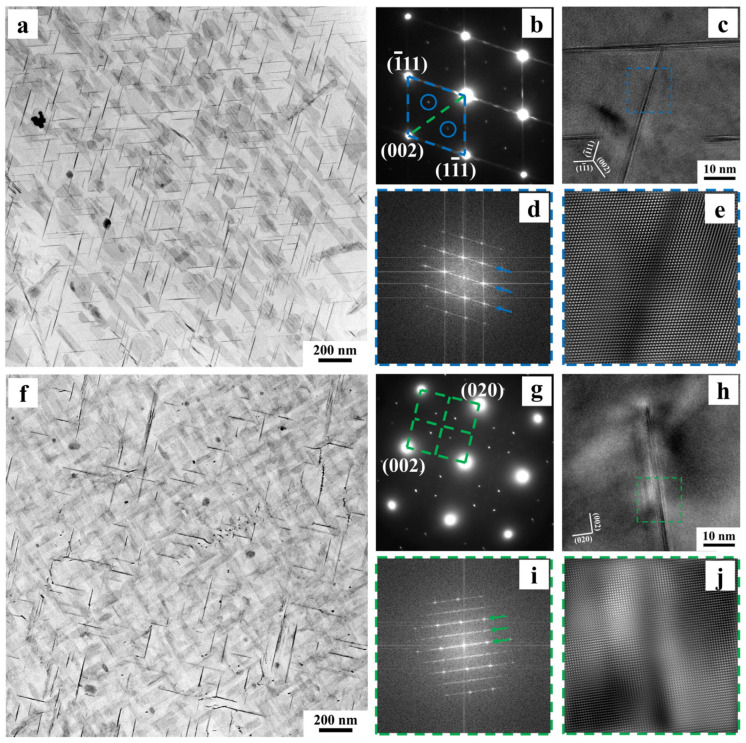
TEM microstructures of the 3.10 Cu alloy after 175 °C/36 h aging treatment along the <110> _Al_ (**a**–**e**) and <100> _Al_ (**f**–**j**) zone axes: (**a**,**f**) bright-field images; (**b**,**g**) corresponding SAED patterns; (**c**,**h**) HRTEM images; (**d**,**e**,**i**,**j**) FFT and IFFT images corresponding to the dashed boxes in (**c**,**h**). The arrows in the FFT patterns of (**d**,**i**) explicitly indicate the fringes corresponding to the phases in (**c**,**h**).

Figure 3 shows TEM images of the 3.10 Cu alloy aged at 175 °C for 36 h along the <110> _Al_ and <100> _Al_ zone axes. Figure 3a,b present bright-field images along the <110> _Al_ zone axis and corresponding selected-area electron diffraction (SAED) patterns. It was observed that the alloy contains a high density of needle-shaped precipitates distributed along two directions, with an angle of approximately 109° between them. As revealed by high-resolution TEM (HRTEM), fast Fourier transform (FFT), and inverse FFT (IFFT) analyses in Figure 3c–e, these precipitates exhibit a semi-coherent relationship with the matrix. Through literature comparisons, these precipitates are identified as disk-shaped T_1_ phase. As the predominant strengthening phase in Al-Cu-Li alloys, it corresponds to the diffraction spots at 1/3{220} and 2/3{220} positions and blue streaks in SAED patterns [7,23]. Due to observation limitations along the <110> _Al_ zone axis, only two of the four variants growing along {111} matrix planes are clearly visible [24]. Correspondingly, Figure 3f–j confirm the presence of a small amount of disk-shaped θ′ phase with a semi-coherent interface in the alloy [25]. Unlike the T_1_ phase, the three variants of θ′ phase develop along {001} planes, enabling clear observation of two mutually perpendicular variants along the <100> _Al_ zone axis [25].

**Figure 4:**
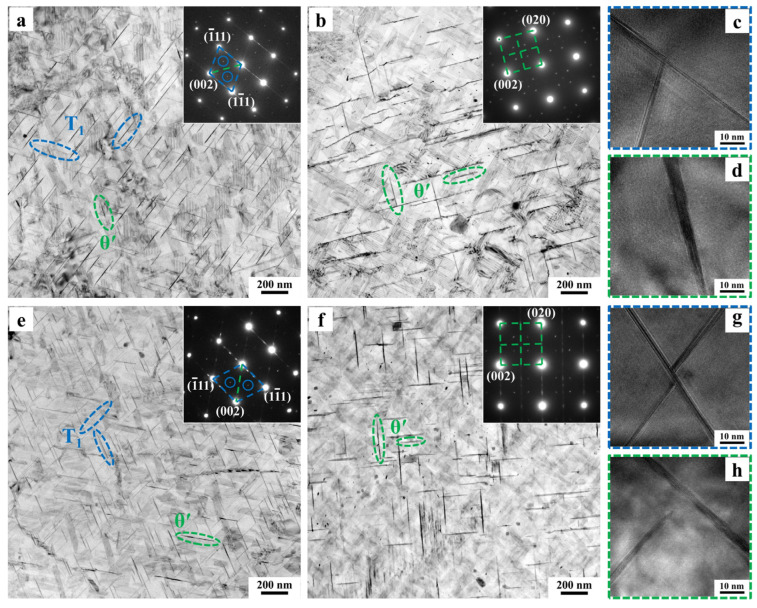
TEM microstructures of the 3.50 Cu alloy (**a**–**d**) and 3.80 Cu alloy (**e**–**h**) after 175 °C/36 h aging treatment along the <110> _Al_ (**a**,**c**,**e**,**g**) and <100> _Al_ (**b**,**d**,**f**,**h**) zone axes: (**a**,**b**,**e**,**f**) bright-field images and corresponding SAED patterns; (**c**,**d**,**g**,**h**) HRTEM images.

Figure 4, analogous to Figure 3, displays TEM microstructures of 3.50 Cu and 3.80 Cu alloys after aging at 175 °C for 36 h along two zone axes. The types of precipitates remain unchanged in these alloys, consisting predominantly of the T_1_ phase and θ′ phase. A comparison analysis of Figure 3a and Figure 4a,e reveals increased density of the T_1_ phase in the 3.50 Cu and 3.80 Cu alloys. Furthermore, the comparative analysis of Figure 3g and Figure 4b,f demonstrates that the diffraction streaks corresponding to the θ′ phase appear most distinctly in the SAED pattern of the 3.80 Cu alloy. Coupled with bright-field observations along the <100> _Al_ zone axis, this confirms the highest quantity of θ′ phase in the 3.80 Cu alloy.

**Figure 5:**
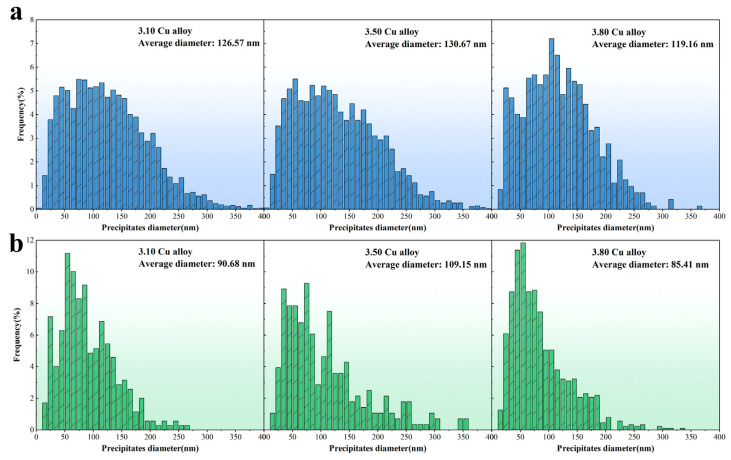
Size distribution of precipitates in the alloys after 175 °C/36 h aging treatment: (**a**) T_1_ phase; (**b**) θ′ phase.

To quantify precipitation variations resulting from Cu content adjustments, statistical analysis of diameter distributions was conducted for both disk-shaped phases, as illustrated in Figure 5. Figure 5a reveals similar diameter distributions of the T_1_ phase in the 3.10 Cu and 3.50 Cu alloys, where approximately 80% of precipitates fall within the 20–200 nm range with comparable proportions per 10 nm interval. In contrast, the 3.80 Cu alloy exhibits a significantly narrower size distribution, with 89% of T_1_ phases concentrated in the 20–200 nm range and the highest proportion observed in the 100–120 nm interval. As quantified in Figure 5a, the average diameters of the T_1_ phase are 126.57 nm, 130.67 nm, and 119.16 nm for the 3.10 Cu, 3.50 Cu, and 3.80 Cu alloys, respectively.

Figure 5b demonstrates that θ′ phases exhibit significantly narrower diameter distributions compared to T_1_ phases, primarily ranging from 20 to 150 nm, with the 3.80 Cu alloy displaying the tightest size distribution. Statistical analysis indicates average θ′ phase diameters of 90.68 nm, 109.15 nm, and 85.41 nm for the 3.10 Cu, 3.50 Cu, and 3.80 Cu alloys, respectively. Combined with bright-field TEM observations, these results suggest that increasing Cu content from 3.10 wt.% to 3.50 wt.% primarily enlarges the diameters of T_1_ and θ′ phases without significant changes in quantity. In comparison, further elevation to 3.80 wt.% promotes increased precipitate density and refined dimensions for both phases. With the increase of Cu content, the enhancement effect on the precipitation of T_1_ and θ′ phases becomes more pronounced, gradually overshadowing the initial promotion effect on precipitate growth.

**Figure 6:**
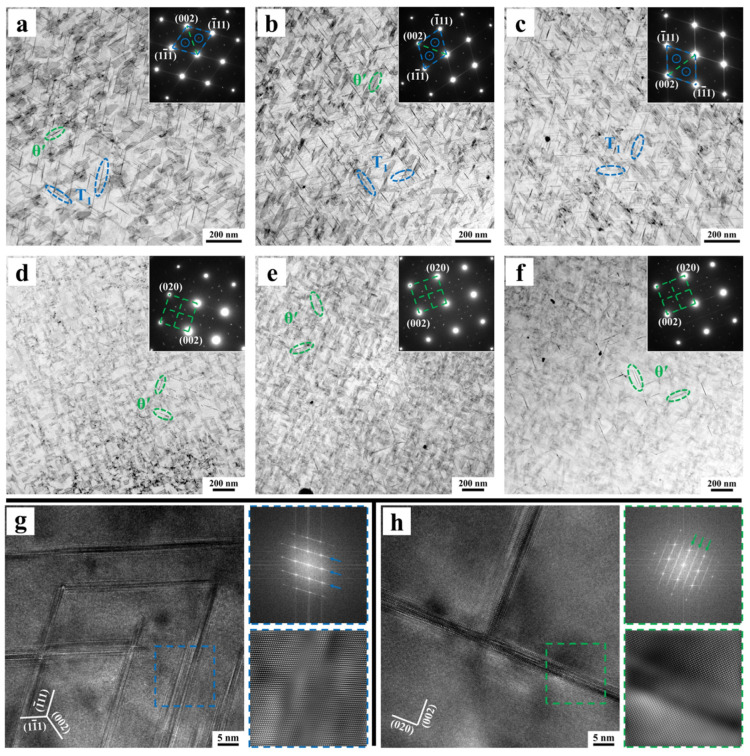
TEM microstructures of the 3.10 Cu alloy (**a**,**d**), 3.50 Cu alloy (**b**,**e**), and 3.80 Cu alloy (**c**,**f**) under 3.5% pre-deformation followed by aging at 155 °C for 36 h along the <110> _Al_ (**a**–**c**,**g**) and <100> _Al_ (**d**–**f**,**h**) zone axes: (**a**–**f**) bright-field images and corresponding SAED patterns; (**g**,**h**) HRTEM, FFT, and IFFT images of T_1_ and θ′ phases in the 3.10 Cu alloy.

Figure 6a–f present bright-field TEM images and corresponding SAED patterns of all three alloys subjected to 3.5% pre-deformation followed by aging at 155 °C for 36 h, while Figure 6g,h display HRTEM analysis of predominant precipitates in the 3.10 Cu alloy. Microstructural characterization confirms that the precipitate types remain unchanged under this aging condition, with T_1_ and θ′ phases as the dominant precipitates. Comparison of Figure 6a–f indicates significantly higher T_1_ phase density and refined dimensions in the 3.50 Cu and 3.80 Cu alloys relative to the 3.10 Cu alloy, while θ′ phase variations require additional quantitative evaluation. Relative to the 175 °C/36 h aging condition, all alloys exhibit substantially increased T_1_ phase density with reduced dimensions, accompanied by decreased size and quantity of θ′ phases.

**Figure 7:**
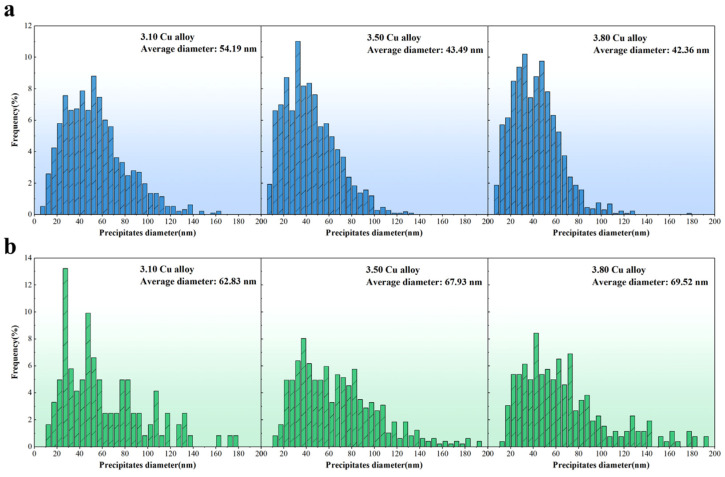
Size distribution of precipitates in the alloys under 3.5% pre-deformation followed by aging at 155 °C for 36 h: (**a**) T_1_ phase; (**b**) θ′ phase.

Figure 7 presents statistical diameter distributions of precipitates under 3.5% pre-deformation followed by aging at 155 °C for 36 h. As shown in Figure 7a, the T_1_ phase diameter distributions of the 3.50 Cu and 3.80 Cu alloys are notably closer compared to the 3.10 Cu alloy, with 80%, 88%, and 91% of T_1_ phases falling within the 10–75 nm range for 3.10 Cu, 3.50 Cu, and 3.80 Cu alloys, respectively. This size confinement directly corresponds to their progressively reduced average diameters (54.19 nm, 43.49 nm, and 42.36 nm). Figure 7b reveals that θ′ phases exhibit less concentrated distributions, requiring a broader 10–95 nm range to encompass ~80% of precipitates. Notably, while the 3.10 Cu alloy displays two distinct θ′ phase concentration peaks (>9% frequency), the average θ′ phase diameters across all three alloys have no significant differences.

Combined with TEM micrographs, these observations confirm that increasing Cu content from 3.10 wt.% to 3.50 wt.% induces significant T_1_ phase refinement and density enhancement, whereas further Cu elevation to 3.80 wt.% yields marginal dimensional changes. Comparative analysis with Figure 5 demonstrates substantial diameter reductions for both phases under 155 °C aging after pre-deformation: T_1_ phases show a 57–67% average diameter decrease, while θ′ phases exhibit a 19–38% reduction. This indicates that variations in aging conditions exert a more pronounced influence on T_1_ phase refinement than θ′ phase.

## 4. Discussion

Experimental results demonstrate that variations in aging conditions and Cu content lead to significant differences in the size and density of T_1_ and θ′ phases, which ultimately govern the mechanical performance of the alloys. However, the relationship between these precipitates and strength remains insufficiently understood, necessitating in-depth quantitative analysis and strengthening calculations. To further quantify the precipitation characteristics of T_1_ and θ′ phases, statistical measurements of precipitate density and thickness were conducted alongside diameter analysis across all three alloys, enabling precise volume fraction calculations. To ensure statistical reliability, over 300 precipitates were analyzed for each condition.

T_1_ and θ′ phases exhibit disk-shaped morphologies, and their volume fractions (*f_V_*) can be calculated using Equations (1)–(3) [24,26,27]:

(1) $$f_{V}=\frac{\pi N_{V}d_{t}^{2}t}{4}$$

(2) $$d_{m}=\left(\frac{t_{s}+\frac{\pi }{4}d_{t}}{t_{s}+d_{t}}\right)d_{t}$$

(3) $$N_{V}=\frac{N}{A_{s}t_{s}}$$

Here, *N_V_* represents the number density of precipitates, *d_t_* denotes the corrected diameter of precipitates, *t* indicates the average precipitate thickness, *d_m_* corresponds to the experimentally measured mean diameter, *t_s_* signifies the sample thickness in the analyzed region, *N* is the total number of counted precipitates, and *A_s_* represents the area of the analyzed region. The determination of *t_s_* requires convergent beam electron diffraction (CBED) experiments using the Kossel–Mollenstedt fringe spacing method [28], which is not detailed here.

Based on the aforementioned methodology, Table 2 presents the quantitative results of precipitate dimensions and volume fractions for alloys aged at 175 °C/36 h. The data reveal that with increasing Cu content, both T_1_ and θ′ phase diameters exhibit an initial increase followed by a subsequent decrease, while thickness variations remain minimal. The T_1_ phase volume fraction demonstrates a monotonic increase, surpassing that of the θ′ phase, with the 3.80 Cu alloy showing a markedly higher θ′ phase volume fraction relative to other alloys. Overall, as Cu content increases from 3.10 wt.% to 3.50 wt.%, the growth of T_1_ and θ′ phases is promoted, accompanied by increased T_1_ phase density. Further elevation of Cu content to 3.80 wt.% substantially enhances the precipitation of both phases while simultaneously suppressing their growth in diameter.

Similarly, the quantitative results of precipitate dimensions and volume fractions under 3.5% pre-deformation followed by aging at 155 °C for 36 h are presented in Table 3. As Cu content increases from 3.10 wt.% to 3.50 wt.%, the T_1_ phase diameter significantly decreases while its volume fraction substantially increases, and the θ′ phase shows moderate growth in both diameter and volume fraction. Further Cu addition leads to minor changes in precipitate diameter, with T_1_ phase thickness and volume fraction slightly decreasing, while θ′ phase thickness and volume fraction exhibit slight increases. Compared to the 175 °C/36 h aging condition, all precipitates exhibit significantly reduced diameters and thicknesses. Notably, while the 3.10 Cu alloy shows a slight decrease in the volume fraction of the T_1_ phase, the other two alloys exhibit significant increases. This indicates that the modified aging conditions strongly promote the precipitation of the T_1_ phase, maintaining high volume fractions despite substantial size refinement.

The competitive precipitation relationship between T_1_ and θ′ phases suggests that extensive formation of the T_1_ phase typically suppresses the nucleation of the θ′ phase, explaining the sharp reduction in the volume fraction of the θ′ phase in low-Cu alloys [9]. However, in high-Cu alloys with sufficient Cu atoms, the precipitation of the θ′ phase becomes more complex, leading to intricate variations in volume fraction compared to the 175 °C/36 h aging condition.

The strengthening effectiveness of precipitates in alloys is influenced by their structural characteristics, coherency relationships with the matrix, and complex morphological features (e.g., diameter, thickness, and volume fraction) [25,29]. To evaluate the strength contributions of T_1_ and θ′ phases, it is essential to clarify their respective strengthening mechanisms.

Previous studies [17,19,24] have demonstrated that the strengthening mechanism of T_1_ phases varies with their size. Smaller T_1_ phases are more susceptible to dislocation shearing, and due to the significant structural differences between T_1_ phases and the matrix, this shearing process generates new interfaces with substantial misfits. To describe this interaction mechanism, Nie and Muddle [30] proposed a precipitation-strengthening model for T_1_ phases, expressed as follows:

(4) $${\Delta \sigma }_{P}=\frac{1.211\;Md_{t}\gamma_{\mathrm{eff}}^{\frac{3}{2}}}{t^{2}}\sqrt{\frac{bf_{V}}{\Gamma }}$$

Here, *M* represents the Taylor factor (3.0 in this study), *d_t_* denotes precipitate diameter, *γ_eff_* is interfacial energy of the T_1_ phase (0.107 J/m^2^), *t* indicates precipitate thickness, *b* corresponds to the Burgers vector (0.286 nm), *f_V_* stands for precipitate volume fraction, and *Γ* represents dislocation line tension, approximately equal to 0.5*Gb*^2^ (where *G* is the shear modulus, ~28 GPa). Based on Equations (1) and (4), ${\Delta \sigma }_{P}\propto {d_{t}}^{2}{N_{V}}^{\frac{1}{2}}t^{-\frac{3}{2}}$, indicating that increases in precipitate diameter and number density enhance strength, with diameter exerting a greater influence, while increased thickness reduces strengthening contributions.

Furthermore, when T_1_ phases exhibit larger thicknesses or diameters, the calculated results from the shearing model significantly exceed experimental observations, with this discrepancy increasing as precipitate dimensions grow [17,29]. To address this phenomenon, researchers [24,29,31] propose that when the diameter or thickness of the T_1_ phase exceeds a critical value, the strengthening mechanism transitions from shearing to bypass. The strengthening increment induced by these larger T_1_ phases can be described by a modified Orowan equation proposed by Zhu and Starke [32]:

(5) $${\Delta \sigma }_{P}=\frac{0.12\;\mathrm{MGb}}{\sqrt{d_{t}t}}\;\left(\sqrt{f_{V}\;}+0.7\sqrt{\frac{d_{t}}{t}}f_{\;V}+0.12\frac{d_{t}}{t}f_{V}^{\;\frac{3}{2}}\right)\;\ln\left(\frac{0.079d_{t}}{b}\right)$$

In contrast to the shearing model, the relationship between precipitate diameter, number density, and strengthening contribution in the bypass model is more complex. Generally, T_1_ phases with 1–3 atomic layers (thickness: 1.15–2.99 nm [33]) exhibit shearable characteristics, while the bypass mechanism becomes more appropriate when the diameter of the T_1_ phase exceeds 100 nm [31,34,35].

As shown in Table 2 and Table 3, all alloys exhibit T_1_ phases with average thicknesses below 2.99 nm but average diameters exceeding 100 nm, necessitating the application of both strengthening models for accurate strength calculations. Based on this analysis, the data in Table 2 were reorganized using 100 nm as the critical diameter, with results presented in Table 4. Under 175 °C/36 h aging, the bypass mechanism dominates T_1_ phase strengthening across all alloys, with shearable phase volume fractions below 0.30%. Increasing Cu content enhances the volume fractions of T_1_ phases governed by both mechanisms. Compared to Table 2, the total volume fraction of the T_1_ phase shows significant improvement, attributed to the higher weighting of diameter in Equation (1). The diameter-based classification increases the calculated volume fraction for bypass mechanisms, thereby elevating the overall volume fraction.

Table 5 presents the reorganized T_1_ phase statistics from Table 3. The data reveal that with increasing Cu content, the diameter of bypass-strengthened T_1_ phases remains relatively stable, while shearable T_1_ phases exhibit slight diameter reduction. The volume fraction of T_1_ phases increases from 2.03% to 3.34% before showing a slight decrease. Compared to the 175 °C/36 h aging condition, all three alloys demonstrate significantly higher volume fractions of shearable T_1_ phases but substantially reduced bypass-strengthened T_1_ phases. Notably, the decreased volume fraction of shearable T_1_ phases in the 3.80 Cu alloy is primarily attributed to reduced diameter and thickness.

Regarding θ′ phases, studies have demonstrated their resistance to shearing deformation [36,37,38]. Nie and Muddle [25] reported no evidence of θ′ phase shearing by dislocations, proposing that the strengthening increment from θ′ phases within the thickness range of 0.6–8.0 nm is governed by the Orowan bypass mechanism. In this work, θ′ phases exhibit average thicknesses ranging from 2.67 to 3.54 nm. Therefore, the strengthening increment induced by θ′ phases can be evaluated using the modified Orowan equation [32]:

(6) $${\Delta \sigma }_{P}=\frac{0.13\;\mathrm{MGb}}{\sqrt{d_{t}t}}\;\left(\sqrt{f_{V}\;}+0.75\sqrt{\frac{d_{t}}{t}}f_{V}+0.14\frac{d_{t}}{t}f_{V}^{\frac{3}{2}}\right)\;\ln\left(\frac{0.079d_{t}}{r_{0}}\right)$$

The internal cutoff radius for dislocation line tension *r*_0_ = 2*b*, while other parameters are defined in the aforementioned equations.

Unlike direct aging at 175 °C/36 h, pre-stretching before 155 °C/36 h aging introduces substantial dislocations, resulting in strain-hardening effects. The yield strength difference between aged and as-quenched states is incorrect as an experimental precipitation-strengthening contribution. Therefore, the strain-hardening increment Δ*σ_dis_* must be calculated prior to precipitation-strengthening evaluation, using the following equation [39]:

(7) $${\Delta \sigma }_{\mathrm{dis}}=M\alpha \mathrm{Gb}\sqrt{\rho }$$

*α* represents the geometric factor (typically 0.2 for Al alloys), and *ρ* denotes the dislocation density. The dislocation density can be calculated from XRD measurements using Equation (8) [40]:

(8) $$\rho =\frac{2\sqrt{3}\varepsilon }{\mathrm{Db}}$$

Here, *ε* and *D* represent lattice strain and grain size, respectively, which can be calculated using the Williamson–Hall (WH) equation [41]:

(9) $$\beta \cos\theta =\frac{K\lambda }{D}+4\varepsilon \sin\theta$$

*β* represents the XRD peak full width at half maximum (FWHM), *θ* is the Bragg angle, *K* denotes the shape factor (typically 0.9), and *λ* is the incident X-ray wavelength (0.1542 nm for Cu Kα radiation).

**Figure 8:**
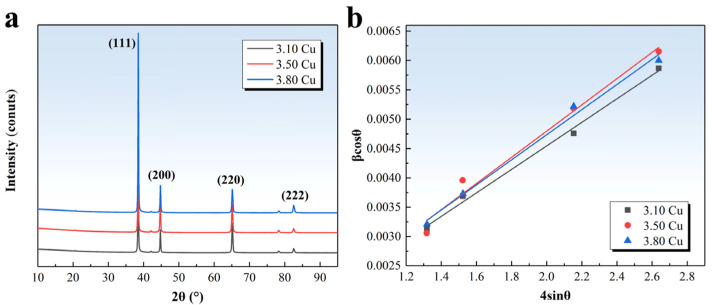
XRD patterns (**a**) and fitted WH plots (**b**) of alloys under 3.5% pre-deformation followed by aging at 155 °C for 36 h.

Figure 8a presents XRD patterns of alloys with varying Cu contents, while Figure 8b shows the fitted curves of *βcosθ* versus 4*sinθ*. Using Equations (8) and (9), the calculated dislocation densities for the 3.10 Cu, 3.50 Cu, and 3.80 Cu alloys are 9.35 × 10^13^ m^−2^, 6.41 × 10^13^ m^−2^, and 8.51 × 10^13^ m^−2^, respectively. Substituting these values into Equation (7) yields corresponding strain-hardening contributions of 46 MPa, 39 Mpa, and 44 MPa. These results indicate substantial strain-hardening effects from dislocations, with minor variations across different Cu contents.

**Figure 9:**
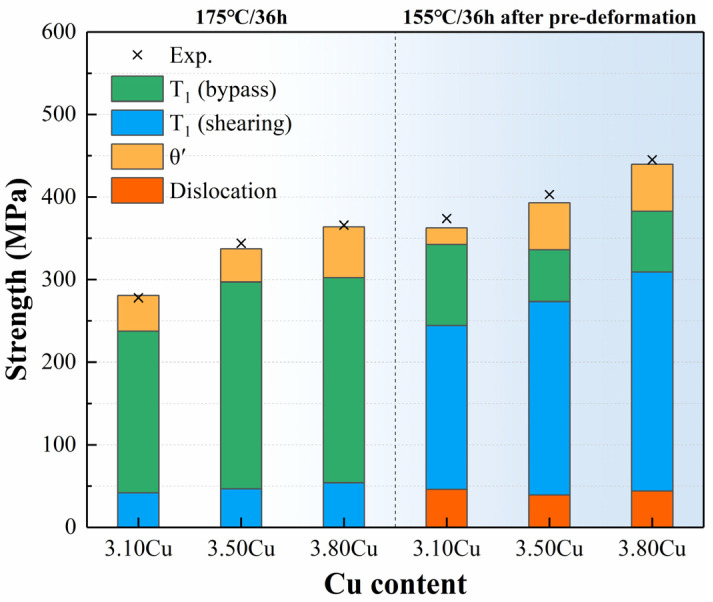
Comparison of calculated and experimental results for precipitate strength contributions in alloys with different Cu contents.

Based on quantitative statistical results and strengthening calculations, the precipitation-strengthening increments for the alloys under different aging conditions were calculated, as shown in Figure 9. The experimental precipitation-strengthening contribution was determined by subtracting the quenched-state strength and strain-hardening contribution from the aged-state yield strength. Notably, strain hardening was included only to obtain experimental precipitation values and is not part of the precipitation-strengthening contribution. The calculated strengthening contributions show good agreement with experimental results for all three alloys. T_1_ phases dominate the precipitation strengthening, accounting for over 83% of the total contribution. θ′ phases contribute 12–17% in most alloys, except for the 3.10 Cu alloy under 3.5% pre-deformation, followed by aging at 155 °C for 36 h, where the contribution is less than 7%.

Under 175 °C/36 h aging, bypass-strengthened T_1_ phases contribute 68–74% of the total strengthening. As Cu content increases from 3.10 wt.% to 3.50 wt.%, bypass-strengthened T_1_ phases drive the strength improvement, while further Cu elevation maintains T_1_ phase contributions but adds ~21 MPa from θ′ phases. For 155 °C/36 h aging after pre-deformation, shearable T_1_ phases dominate, contributing 84–94% of the total strengthening increment. Increasing Cu content from 3.10 wt.% to 3.50 wt.% reduces bypass contributions while enhancing shearable T_1_ phases and θ′ phases, resulting in a ~37 MPa strength increase. Further Cu elevation maintains θ′ phase contributions but increases both strengthening mechanisms of T_1_ phases, yielding an additional ~40 MPa improvement.

Comparative analysis reveals a transition in the dominant strengthening mechanism of T_1_ phases between aging conditions. At higher Cu contents, shearable T_1_ phases provide greater strengthening than bypass mechanisms, indicating that aging conditions enhance the refinement effect of Cu on T_1_ phases, ultimately leading to significantly improved strength in high-Cu alloys.

## 5. Conclusions

In the present study, the precipitation behavior and mechanical properties of Al-Cu-Li alloys with varying Cu contents under different aging treatment conditions were systematically investigated, with quantitative analysis methods employed to elucidate the correlation between precipitates and strengthening mechanisms. The principal conclusions are summarized as follows:

(1) Increased Cu content accelerated the hardening rate during initial aging stages while enhancing peak hardness and strength. Under 3.5% pre-deformation followed by aging at 155 °C, hardness and strength exhibited significant improvement, with a markedly greater disparity between the 3.80 Cu and 3.50 Cu alloys in these properties;
(2) Under 175 °C/36 h aging conditions, the primary precipitates consisted of abundant T_1_ phases and minor θ′ phases. With the increase of Cu content, the enhancement effect on the precipitation of T_1_ and θ′ phases becomes more pronounced, gradually overshadowing the initial promotion effect on precipitate growth;
(3) For pre-deformed samples aged at 155 °C/36 h, while maintaining identical precipitate types, significant refinement in precipitate diameter and thickness was observed alongside remarkable T_1_ phase proliferation. Elevated Cu content induced continuous reduction in T_1_ phase dimensions while promoting θ′ phase precipitation with slight diameter enlargement;
(4) Comparative analysis revealed that the strengthening mechanism of the T_1_ phase transitioned from bypass-dominated to shearing-dominated when comparing 175 °C/36 h with 155 °C/36 h aging after pre-deformation. Concurrently, enhanced Cu-induced refinement effects on T_1_ phases contributed to substantially improved strength in high-Cu alloys under the latter condition.

## Figures and Tables

**Table 1:** Chemical composition of the Al-Cu-Li alloys (wt.%).

Alloy	Cu	Li	Mg	Ag	Zn	Mn	Zr	Al
3.10 Cu	3.14	0.84	0.33	0.36	0.12	0.42	0.09	Bal.
3.50 Cu	3.54	0.88	0.37	0.39	0.13	0.41	0.13	Bal.
3.80 Cu	3.76	0.82	0.34	0.38	0.12	0.42	0.09	Bal.

**Table 2:** Quantitative data for precipitates of alloys after 175 °C/36 h aging treatment.

Alloy	3.10 Cu		3.50 Cu		3.80 Cu	
Phase	T_1_	θ′	T_1_	θ′	T_1_	θ′
*d_t_* (nm)	139.39	97.93	147.49	121.79	132.07	92.78
*t* (nm)	1.96	3.54	1.93	3.45	1.98	3.34
*f_V_* (%)	1.88	0.53	2.42	0.47	2.63	0.88

**Table 3:** Quantitative data for precipitates of alloys under 3.5% pre-deformation followed by aging at 155 °C for 36 h.

Alloy	3.10 Cu		3.50 Cu		3.80 Cu	
Phase	T_1_	θ′	T_1_	θ′	T_1_	θ′
*d_t_* (nm)	58.47	68.36	48.51	77.97	46.85	79.36
*t* (nm)	1.42	2.73	1.49	2.67	1.34	3.15
*f_V_* (%)	1.80	0.11	3.20	0.63	2.94	0.74

**Table 4:** Quantitative data for T_1_ precipitates of alloys after 175 °C/36 h aging treatment.

Alloy	3.10 Cu		3.50 Cu		3.80 Cu	
Phase	T_1_ (bypass)	T_1_ (shearing)	T_1_ (bypass)	T_1_ (shearing)	T_1_ (bypass)	T_1_ (shearing)
*d_t_* (nm)	193.02	63.51	203.70	64.15	179.70	64.98
*t* (nm)	1.96	1.96	1.93	1.93	1.98	1.98
*f_V_* (%)	2.14	0.16	2.79	0.18	2.88	0.26
	2.30		2.97		3.14	

**Table 5:** Quantitative data for T_1_ precipitates of alloys under 3.5% pre-deformation followed by aging at 155 °C for 36 h.

Alloy	3.10 Cu		3.50 Cu		3.80 Cu	
Phase	T_1_ (bypass)	T_1_ (shearing)	T_1_ (bypass)	T_1_ (shearing)	T_1_ (bypass)	T_1_ (shearing)
*d_t_* (nm)	135.63	53.19	133.25	47.29	134.85	45.43
*t* (nm)	1.42	1.42	1.49	1.49	1.34	1.34
*f_V_* (%)	0.64	1.39	0.35	2.99	0.40	2.72
	2.03		3.34		3.12	

## Data Availability

The original contributions presented in this study are included in the article. Further inquiries can be directed to the corresponding authors.
